# Readiness for voice assistants to support healthcare delivery during a health crisis and pandemic

**DOI:** 10.1038/s41746-020-00332-0

**Published:** 2020-09-16

**Authors:** Emre Sezgin, Yungui Huang, Ujjwal Ramtekkar, Simon Lin

**Affiliations:** Nationwide Children’s Hospital, 700 Children’s Drive, Columbus, OH 43205 USA

**Keywords:** Health policy, Public health

## Abstract

To prevent the spread of COVID-19 and to continue responding to healthcare needs, hospitals are rapidly adopting telehealth and other digital health tools to deliver care remotely. Intelligent conversational agents and virtual assistants, such as chatbots and voice assistants, have been utilized to augment health service capacity to screen symptoms, deliver healthcare information, and reduce exposure. In this commentary, we examined the state of voice assistants (e.g., Google Assistant, Apple Siri, Amazon Alexa) as an emerging tool for remote healthcare delivery service and discussed the readiness of the health system and technology providers to adapt voice assistants as an alternative healthcare delivery modality during a health crisis and pandemic.

## Introduction

With the rapid spread of the deadly Corona Virus Disease of 2019 (COVID-19) outbreak, many countries faced a demand surge for inpatient and intensive care unit beds and the associated healthcare services and resources. The health systems have also been under tremendous stress to meet the unprecedented healthcare demand while maintaining inpatient and outpatient services for other healthcare needs. This unexpected public health crisis with sustained spread has magnified the problems that health systems encounter when relying on the face-to-face care delivery model almost exclusively. The motivation to reduce the spread, continue providing care, and contain operational costs forced the rapid deployment of telehealth.

The timely actions taken on by federal agencies in response to the COVID-19 emergency to relax regulation and privacy enforcement, as well as to expand allowable reimbursement, were critical factors for telehealth’s implementation^1,2^. One of the early congressional decisions was H.R. 6074: “Coronavirus Preparedness and Response Supplemental Appropriations Act, 2020”, which allowed Health and Human Services (HHS) to waive several Medicare restrictions and requirements temporarily regarding telehealth services during the COVID-19 emergency^2^.

The use of interactive two-way video teleconferencing in medical consultation mitigated the volume of non-essential in-person visits at hospitals, preserved critical resources and supplies needed for treating COVID-19 patients, and reduced exposure for healthcare professionals and patients^3^. Moreover, other digital health tools had a significant role in the fight against COVID-19. Intelligent conversational agents and virtual assistants have proven their potential to serve as an intermediary to reduce the burden in the healthcare system for monitoring and consulting the public^4^, through their versatility, accessibility, and scalability for naturalistic communications with the end-users^5^. Chatbots and voice assistants (VA) are popular examples of intelligent conversational agents and virtual assistants. In the literature, the definition and implementation of VA and chatbots are intertwined. Both enable communications with end-users via natural language, achieved through rule-based dialog or machine learning^6^. Both may include multimodal interaction support (e.g., screen, text, speech, and sound), with VAs primarily engaging users through voice interface and chatbots through text. Generally, chatbots are perceived to be service-specific conversational agents that typically engage in multi-turn dialogues (e.g., Woebot- a therapy chatbot), while VAs are perceived to be virtual assistants which need voice commands to interact and complete tasks (e.g., Amazon Alexa and Alexa skills)^7^.

Intelligent conversational agents and virtual assistants have been used and tested for responding to health information seeking activities^8–12^. Recent efforts have expanded their utilities in health assessment. The Centers for Disease Control and Preventions (CDC) and Microsoft joined forces to create a COVID-19 text-based chatbot using reliable and evidence-based information in self-assessment to eliminate basic information seeking^13^. The World Health Organization (WHO) released a text-based chatbot via a highly popular messaging app, WhatsApp, to respond to public questions about COVID-19^14^. Apple released an app to share COVID-19 information and updates using CDC resources, which can also be accessible through Siri (Apple’s VA)^15^. Amazon released Alexa VA features to help users setup routines during staying at home and providing tips, information, and guidance about COVID-19^16^. Frameworks for COVID-19 screening with chatbots have been proposed and implemented at healthcare institutions^17,18^.

Despite these promising efforts in response to the pandemic, questions still remain. “Are the healthcare systems ready to adopt conversational agents and virtual assistants to be used in response to a pandemic?” and “Are technology providers ready to collaborate and support healthcare communications?” In this commentary, the rest of our discussions focus on VA, which holds a significant share in the intelligent conversational agents and virtual assistants market in terms of accessibility and consumer adoption^19^. We comment on four aspects of VA adoption in healthcare: (1) the current state of voice assistants, (2) the readiness of the health system, (3) the readiness of the technology providers, and (4) the impact of VA in post-COVID health delivery.

## Current state of voice assistants

Arguably the popularity with consumer VAs might have started with the launch of Apple Siri in 2011. After Amazon introduced Alexa VA and Echo devices in 2014, the technology around speech recognition, text-to-speech and speech-to-text, natural language processing methods, and conversational AI have been continuously improving as the number of VA users have been increasing^19,20^. VAs have been adopted by healthcare institutions to respond to health information seeking users by providing healthcare tips and guidelines (Cleveland Clinic’s Tip of the Day^21^, Boston Children’s KidsMD^22^), health news (Mayo Clinic’s news network^23^), updates about hospital operations, communication and navigation (Ohio Health^24^, New Hanover Regional Medical Center^25^), first aid guidance (Mayo Clinic’s first aid^26^, American Red Cross’ first aid^27^), and medical communications (Boston Children’s My Children’s Enhanced Recovery After Surgery^28^). The convenience of natural conversation and hands-free interaction through digital devices has the potential to improve the effectiveness of health information delivery and communication^7,19^.

However, limitations and concerns still exist about VA use in practice. Some limitations are the need for persistent internet connection, deficiencies in speech recognition and comprehension, the need for IT infrastructure for health system integration [e.g., electronic health record (EHR)], and Health Insurance Portability and Accountability Act (HIPAA) compliance to exchange personal health information^7,29^. In 2019, Amazon initiated HIPAA compliant VA services (“Alexa skills”)^30^, yet, the implementations were limited to only some services and organizations. Also, the privacy of personal information and the possibility of continuous listening mode raised major concerns^19^.

## Readiness of the health system

In response to the pandemic, HHS announced that non-compliance with HIPAA for non-public facing communication products (such as popular audio/video communication apps) might not be penalized in the setting of healthcare providers providing telehealth services in the current emergency conditions^31^. The Coronavirus Aid, Relief, and Economic Security (CARES) Act and the Federal Communications Commission (FCC) Telehealth Program issued additional funding for providers to enable remote care services for patients through digital communication services^1^. Also, the Food and Drug Administration (FDA) announced that they would not enforce requirements of the Federal Food, Drug, and Cosmetic Act (FD&C Act) for low-risk non-medical device software and mobile apps, which can potentially facilitate responding to COVID-19^32^. These relaxations may have enabled technology providers and healthcare organizations the ability to react quickly with VA-based healthcare communication and services.

However, current VA and other intelligent, conversational agent implementations showed the disconnect with public health authorities. Many VA solutions failed to present up-to-date and reliable information^33^, which may cause misinformation dissemination^34^. One measure Google has taken to prevent misinformation was to block third-party developers adding VA apps for the Google Assistant about the pandemic^35^. Such control can ensure the distribution of reliable pandemic information. Still, the added control could severely curtail information delivery effectiveness and reduce the availability of potentially useful VA apps for the end-users. Therefore, an emergency digital health and VA use action plan for health systems is necessary to mitigate any risks that can arise from lowering central control to deliver health services through VAs. The plan should also provide metrics and evaluation guidelines for controlling misinformation over the VA contents offered by technology providers and third-party developers.

In contrast to telehealth practice that requires human providers, VAs could be autonomous as they can leverage AI to gather and share information on the pandemic (public health level) as well as provide personal health feedback (personalized health level). The relationship between telehealth and VA-based healthcare delivery needs to be distinguished clearly. The next step in establishing the regulatory ground rules in telehealth (e.g., Creating Opportunities Now for Necessary and Effective Care Technologies (CONNECT) for Health Act^36^) should be updated regarding VA as part of a health information delivery system. Even though current health information privacy regulations (e.g., HIPAA) could be used to regulate VAs, they may fall short in covering new technologies and implementations that limit the utility of these technologies^37^. Since the current telehealth structure has not been optimized and is continuously being improved, discussing VA’s role in supporting care delivery and making it part of existing plans, regulations, and infrastructure for telehealth is essential.

The information system (IS) infrastructure requires updating to support the digital innovation of the health system, accommodate the new workflow of health information, and ensure privacy and security. First of all, the VA needs to connect electronic health records (EHRs) through interoperable application programming interfaces (APIs). EHR connection will allow AI to tailor general guidelines of the pandemic using personal health information from the EHRs to provide personalized advice or predict outcomes for public health. Keesara et al.^38^ suggested the IS infrastructure could be developed using existing service models, such as Software as a Service (SaaS). As such, federal and state regulations on health data management and control (e.g., HIPAA), as well as hospital command system protocols, need updating to include the flexibility necessary to accommodate the infrastructure for VA utilization.

## Readiness of technology providers

**Fig. 1:**
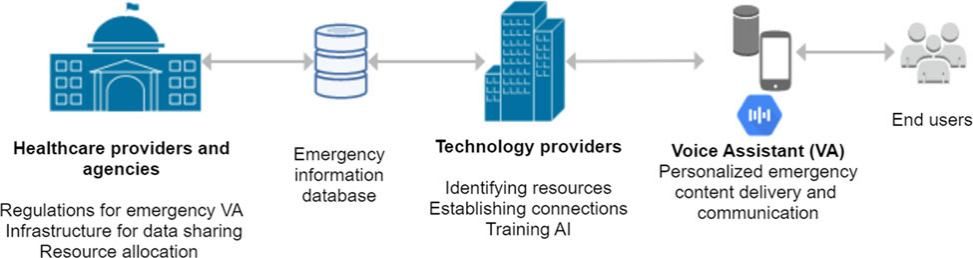
Voice assistant (VA) use for emergency information delivery. Healthcare providers and agencies support with the regulations, infrastructure and resources to be shared with technology providers. Technology providers identify the resources and connect to retrieve up-to-date information over an emergency information database, which is used to inform end-users via VA while using AI to personalize the content.

As aforementioned, communication between public health authorities and technology providers is necessary. Specifically, VA developers should be a primary stakeholder in developing the emergency digital health and VA use action plan to ensure their readiness for rapid deployment and assistance of VA solutions in case of healthcare emergencies. Therefore, an emergency voice assistant service to support healthcare delivery and information dissemination should be strategized. The proposal is to have VAs gather up-to-date information from designated resource channels maintained by health authorities. Strategic approaches should include establishing connections with federal, state, and local government health agencies, establishing agreements and contracts for compliant services, creating data flow channels, and potentially training AI to synthesize information to deliver and communicate personalized content optimally (Fig. 1). Segregating information resources and distributing in state-level to city-, county- and hospital-levels could ensure the relevance and validity of the information. Box 1 depicts a potential VA use scenario for Ohio using the proposed framework.

As the pandemic has put the public under restricted and isolated living conditions for a sustained period, technology providers should reconsider how to engage with health information seeking end-users, who could be a caregiver, provider, or a chronically ill patient. A new perspective on voice-user interface design could be necessary for VA to engage with end-users with various healthcare needs during a crisis to communicate and exchange health information effectively. At-risk communities who can potentially benefit from VA, such as the elderly, should especially be considered^39,40^. Currently, VAs’ user engagement and comprehension may not be optimal with medical communications (e.g., not understanding a command, providing erroneous or misleading content)^41,42^. The public would be better served if new designs would mitigate the risk of errors and user frustration as well as building comfort and trust in medical conversations.

VA should also be context-aware and have an effective fallback strategy in content delivery. For example, failure in understanding a full question about COVID-19 should be remedied by referring to the most relevant public health resource or information channel by intelligently inferring from the context. Since VAs can be synchronous, asynchronous, and multimodal (voice-only vs. voice and visual, over the PC, smart speaker, or mobile phone), technology providers could also strategize the delivery of health content based on the receiver’s socioeconomic status or digital ecosystem^43^. For instance, voice-only content delivery over a smart speaker could be preferable for patients with low literacy or physically unable to read or engage with devices. Yet, a VA over a phone line could be better in rural areas limited by broadband Internet access or quality.

Box 1. User scenario of VA use in disseminating pandemic updates in Ohio Potential VA use in disseminating pandemic updates in Ohio

Ohio Department of Health resources^a^ provide up-to-date information on the state-wide update of state orders and pandemic cases; Columbus city resources update metropolitan area residents with city community resources transportation under emergency^b^; Franklin County provides guidelines and updates on how to access local supplies and resources under emergency^c^; Nationwide Children’s Hospital resources provide updates on patient health supplies, screening and visitation regulation^d^. However, these resources should be optimized for VA use, and could be distributed with a parallel channel to technology providers, e.g., emergency information database.

^a^ https://coronavirus.ohio.gov/wps/portal/gov/covid-19/home

^b^ https://www.columbus.gov/covid19resources/

^c^ https://covid-19.myfcph.org/

^d^ https://www.nationwidechildrens.org/family-resources-education/health-wellness-and-safety-resources/covid-19

## Impact of VA in the post-COVID health delivery

In addition to providing reliable, relevant, and up-to-date COVID-19 information and guidelines to the public, VA can have conversations with patients who need routine care with healthcare communication (e.g., triaging, screening, asking standard exam questions, receiving questions for providers, providing medication guidelines). Such implementation would reduce the impact of delayed care and decrease dependency on providers on routine tasks. According to a McKinsey survey report published in March 2020, one in every three survey respondents (*n* = 979) canceled medical appointments and planned to reschedule after the pandemic subsides^44^. These responses would suggest a delayed increase in the demand for healthcare. Therefore, healthcare providers need to plan for employing VA services for continuous remote healthcare delivery. In that regard, the delivery of periodic pandemic updates could be the short-term goal. In contrast, conversational assistance in health screening, remote monitoring, and communication support could be the long-term goal. One potential implementation could be transferring the health screening and monitoring process to VA through EHR-integrated services (e.g., speech-to-text message delivery on the patient portal), which would give healthcare providers the flexibility with an asynchronous response to patient needs. The feasibility of EHR-integrated VA in the hospital setting showed promise in patient-facing bedside assistance^45^.

On the other side, in hospital operations, VA could facilitate the control for infection and reduce the hands-on documentation burden by assisting physicians with dictating visit notes, ordering tests, and charting or navigating EHR hands-free^46^. VA could assist the nurse triaging by assessing the risk level of the patients through conversational assessment, as proposed with chatbots^47^. In a broader scale, VA could also utilize voice as a digital biomarker, which could be leveraged for the continuous screening and detection of pandemic symptoms, such as identifying respiratory disorders^48,49^.

## Conclusion

As telehealth is playing a significant role in healthcare, VA should be considered as a supportive tool in multimodal digital solutions during a pandemic crisis. The readiness of healthcare systems, infrastructure, and technology providers are essential to build VAs in response to pandemic and support healthcare delivery while aiming to mitigate the risk of the disease spread and reduce the stress on the health system. Readiness would require healthcare experts to partner with technology developers, regulators, and legislators as well as providing guidelines and framework for implementation. The WHO’s recent effort demonstrates an excellent example of an information management framework during a pandemic^50^. A coordinated effort and -stakeholder strategic planning are necessary to improve future implementations and adoptions of VAs in the healthcare domain and expand its assistance beyond information delivery to health assessment^8^. Also, the effectiveness of VA in healthcare delivery during a health crisis requires further studies to assess fully.
